# Gaining insight into the views of outpatients with Huntington’s disease regarding their future and the way they deal with their poor prognosis: a qualitative study

**DOI:** 10.1186/s12904-020-00706-x

**Published:** 2021-01-12

**Authors:** Marina R. Ekkel, Marja F. I. A. Depla, Els M. L. Verschuur, Ruth B. Veenhuizen, Cees M. P. M. Hertogh, Bregje D. Onwuteaka-Philipsen

**Affiliations:** 1 Department of Medicine for Older People, Amsterdam Public Health Research Institute (APH), Amsterdam UMC, Vrije Universiteit Amsterdam, PO Box 7057, 1007 MB Amsterdam, The Netherlands; 2 School of Health Studies, HAN University of Applied Sciences, Nijmegen, The Netherlands; 3 Huntington Expert Centre Atlant, Apeldoorn, The Netherlands; 4 Department of Public and Occupational Health, Amsterdam Public Health Research Institute (APH), Amsterdam UMC, Vrije Universiteit Amsterdam, Amsterdam, The Netherlands

**Keywords:** Advance care planning, Huntington’s disease, Qualitative research, Neurodegenerative disease, Patients’ perspectives

## Abstract

**Background:**

Huntington’s disease (HD) has a poor prognosis. Decision-making capacity and communication ability may become lost as the disease progresses. Therefore, HD patients are encouraged to engage in advance care planning (ACP). To improve ACP for HD patients, there is a need to better understand how these patients face their poor prognosis.

**Aim:**

To gain insight into the views of HD patients who receive outpatient care regarding their future and the way they deal with the poor prognosis of their disease.

**Methods:**

A qualitative study using semi-structured interviews with 12 patients with HD (7 outpatient clinic, 3 day care, 2 assisted living facility). Audio-recorded interviews were transcribed verbatim. Through reading and re-reading interviews, writing memos and discussions in the research team, strategies were identified.

**Results:**

Three strategies emerged for facing a future with HD. Participants saw the future: 1) as a period that you have to prepare for; 2) as a period that you would rather not think about; 3) as a period that you do not have to worry about yet. Participants could adopt more than one strategy at a time. Even though participants realized that they would deteriorate and would need more care in the future, they tried to keep this knowledge ‘at a distance’, with the motivation of keeping daily life as manageable as possible.

**Conclusions:**

Official ACP guidelines recommend discussing goals and preferences for future treatment and care, but patients tend to want to live in the present. Further research is needed to elucidate the best approach to deal with this discrepancy.

**Supplementary Information:**

The online version contains supplementary material available at 10.1186/s12904-020-00706-x.

## Background

Huntington’s disease (HD) is an autosomal dominant neurodegenerative disease [1]. Typically, HD is diagnosed around age 30–50, and it has a clinical course of 17–20 years until death [1]. Patients with HD experience complex and unpredictable changes in their physical, cognitive, emotional and behavioral functioning. These symptoms lead to a decline in functional capacity and loss of independence, which require nursing care in the more advanced stages [2]. At present, there is no cure for HD.

In HD, decision-making capacity and communication ability may become impaired or lost as the disease progresses. Therefore, patients are encouraged to engage in advance care planning (ACP) and draw up an advance directive early in the course of HD [3–5]. ACP is defined as the ability to enable individuals to define goals and preferences for future medical treatment and care, to discuss these with family and health-care providers, and to record and review these preferences if appropriate [6]. There is evidence that ACP positively impacts the quality of end-of-life care [7]. In general, advance directives concern treatment preferences, but in the Netherlands they may also concern a request for euthanasia or physician assisted suicide (PAS), in line with the Euthanasia act of 2002 [8].

There has been some research on the views of patients with neurodegenerative diseases on planning future care and being engaged in ACP. It has been reported that patients vary in their ability to speak with others about their condition and prognosis [9], and that patients find it difficult to see what kind of support they would need in the future [10]. Furthermore, Clarke et al. [9] found that some patients wished to plan extensively for the future, while others preferred to take each day as it comes. That finding is similar to the findings of De Boer et al. [11], who showed that overall, patients with Alzheimer’s disease live one day at a time and avoid worrying about the future.

Even though HD shares characteristics with other neurodegenerative diseases, like the progressive nature, the uncertainty of the disease course, and its incurability [12, 13], there are also important differences that could influence the way patients with HD look at their future and future care. Next to a relatively long disease trajectory [3, 14, 15] and a younger age of onset [14], the inherited nature of HD imposes a unique burden on patients with HD [3, 14–17]. Each child of an affected parent is at 50% risk of becoming affected in later life by HD [1]. This means that patients generally have witnessed an HD disease trajectory in their family. Having experienced the consequences of HD, and thereby having the feeling that they have potentially seen their own future, the views of HD patients on future care and ACP may be unique for the neurodegenerative diseases population. A Dutch survey found that having thoughts or wishes about the end of life was only related to being familiar with HD in the family, and not to other demographic or clinical variables, like age, education, or disease stage [18].

Because of these unique characteristics of HD there is a need for a better understanding of the way these patients think about their future. Therefore, the aim of this study is to gain insight into the views of HD patients regarding their future and the way they deal with the poor prognosis of their disease. These insights are relevant in supporting ACP with patients with HD.

## Methods

### Study design

Semi-structured interviews were carried out with HD patients who received outpatient care, to invite and encourage them to share their views and perspectives on a future with HD [19, 20].

### Setting and participants

Participants were recruited from four Dutch nursing homes that provide specialized outpatient care to patients with HD. To be included in the study, patients had to be able to understand the goals of the study, to speak comprehensively in Dutch and to give informed consent. To obtain variation in the potential factors influencing our research question, a purposive sampling strategy was used to maximize variety in the sampling of participants by gender, age, disease stage, and family living conditions [21]. Inclusion ended due to pragmatic considerations (difficulty to include participants and time restraints).

### Procedure

Nurses who were involved in outpatient care judged which patients would be suitable for participating, according to the aforementioned criteria. They informed the patients about the study and asked whether the researcher (ME) could approach them for inclusion. Subsequently, participants were approached by the researcher and informed about the study verbally and through an information letter. Informed consent was given before the interview. The interviews were carried out face to face at a time and place convenient to the participants (usually their homes). The interviews took place between August 2017 and July 2019 and lasted between 32 and 67 min. Participants were interviewed individually, however in two interviews a health-care professional was present on request of the patient. This caregiver was asked not to contribute and any comments she made were not used for the data analysis.

### Data collection

After examination of relevant literature (e.g. [11, 18]), a topic list was formulated that was used as a guide during the interviews (see Supplementary file 1). The interviews focused on 1) thoughts and attitudes towards the future, future care and the end of life, and 2) discussing these topics with family members and health-care professionals. Questions were not asked in a fixed order, but followed up on the answers participants provided. All interviews were conducted by the first author (ME). As not all aspects of the interviews would be apparent in the transcript, like for example the amount of choreatic movement, the level of dysarthria or the rapport in the interview, field notes were kept which described reflections on the interviews [22, 23]. The interviews were audio-recorded, transcribed verbatim, and all personal identifiers were removed.

### Data analysis

First, the transcripts were read and re-read by ME, MD and BO in order to become familiar with the data. Memos were written about every transcript to enable meaning to be extracted from the data and to achieve abstraction while remaining true to the data [22]. Second, the memos were compared and discussed by ME, MD and BO and three periods of ‘awareness of the consequences of HD’ could be distinguished: 1) the past, the period of predictive testing; 2) the present, being aware of current symptoms of HD; and 3) the future, realizing the poor prognosis of HD. Third, ME structured excerpts from the interviews according to a framework consisting of these three periods. Then, these periods were discussed by three researchers (ME, MD, BO). First on the participant level, to get a full picture of the way the participant faces the consequences of his or her disease; and next exclusively in relation to the future, as this was most relevant to the research question. In this study we will focus on the future time period. Three strategies for dealing with the consequences of HD in the future were identified. These were checked against all transcripts. Finally, these strategies were discussed in the research team (ME, MD, BO, RV, EV, CH) and consensus was reached on their definition and meaning.

### Ethical issues

All participants provided written informed consent. It was emphasized that their opinions would be dealt with confidentially and would not be provided to the physician or other health-care professionals. The Medical Ethics Committee of the VU University Medical Center reviewed this study protocol and concluded that the Medical Research Involving Human Subject Act (WMO) did not apply to this study. Therefore, an official approval of this study by the committee was not required (VUmc METc 2017.218).

## Results

### Study sample

Twelve patients participated in the study. Table 1 shows their demographics. All patients visited the outpatient clinic one or two times a year at the time of inclusion. However, one patient was admitted to a nursing home between inclusion and the first interview and would therefore no longer visit the outpatient clinic from that moment. Another patient lived independently in an assisted living facility, where help would be stand-by when needed. This patient continued to visit the outpatient clinic. Three patients visited day care several days a week. Four out of twelve patients were unfamiliar with HD before they were diagnosed. From the eight patients who knew they were at risk of HD, four chose to undergo predictive testing. One patient claimed that she did not have HD yet and experienced no symptoms, even though she received HD-related care for several years.

**Table 1:** Demographic details of the participants

Demographics	Participants
Female (n, %)	8 (67%)
Age in years (mean, range)	52 (27 to 80)
Married or living with partner (n, %)	8 (67%)
Number of children (mean, range)	2.3 (0 to 6)
Health care use (n, %)	
Outpatient clinic	7 (58%)
Day care and outpatient clinic	3 (25%)
Assisted living facility and outpatient clinic	1 (8%)
Nursing home (without outpatient clinic)	1 (8%)
Familiar with HD before they were diagnosed (n, %)	8 (67%)
Chose to undergo predictive genetic testing (n, %)	4 (50%)
Time since predictive genetic testing (according to patient) in years (mean, range)	14 (10 to 25)
Diagnosed with HD (according to patient) (n, %)	11 (92%)
Time since diagnosis (according to patient) in years (mean, range)	5.4 (0.2 to 15)

### Thinking about the future

All participants seemed to be aware of their poor prognosis. They were afraid that their functioning would decline, that they would have to be admitted to a nursing home, or that they would no longer be able to take care of their children or other loved ones. Several sources of information were mentioned when it came to shaping their future image. Participants who had experienced HD in their family members spoke about these experiences in the interview. Participants who were unfamiliar with HD before their diagnosis talked about seeing other patients on the internet, at the outpatient clinic, or at the day care centre.

### Identified strategies

When it came to facing the future with HD, three strategies emerged from the interviews. Participants saw the future: 1) as a period that you have to prepare for; 2) as a period that you would rather not think about; 3) as a period that you do not have to worry about yet.

#### The future is something that you have to prepare for

In this strategy participants wanted to prepare for their poor prognosis by 1) thinking about the necessary care in the future; or 2) drafting up an advance euthanasia request. After thinking about the future and making preparations, participants returned to living their daily lives.

#### Thinking about future care

One way of dealing with the future was thinking about the care participants were going to need in the future. Sometimes, they talked about this with their family and professional caregivers. However, these thoughts were not very elaborated nor widespread; the wishes for care in the future were mainly an extension of the presently enabled care.

#### Making arrangements for euthanasia

Euthanasia was a topic that several participants spontaneously mentioned during the interviews. Characteristic for this strategy was that participants prepared for their future by specifying the conditions when they no longer found that future bearable. As a result, the poor prognosis was given a concrete representation. Sometimes that representation was a threatening image that the participant never wanted to endure, like *“drooling in a wheelchair”* [P3] or having *“that hollow empty look”* [P8]. Other participants gave a more elaborate description of symptoms and consequences that made up their advance euthanasia request, like the following participant:

P6*: “I am always creative and working with my hands. Suppose that at some point I can’t do that anymore, that could also be a consideration for me, [...], to say I’m done. [ …*] *Dementia. That’s another thing that makes me think: thanks but no thanks [laughs]. [...] Look, if, for example, my mind is still clear, you know, and I can still use my hands, but I would, for example, be fed through a feeding tube, then that wouldn’t be a big problem at all. [...] No, if that meant I could function OK, then I think: fine. [...] Look, even if it meant I wouldn’t be able to talk very well, but I could use a computer or something, well, fine. Look, I’d still be able to communicate. But if I can’t do that anymore, so not be able to convey to others what I want, what I mean, and not be able to express myself, I would really hate that.”*

For some participants, like P6, having an advance euthanasia request meant that they could carry on with their daily lives. For others, the advance euthanasia request did not bring this peace of mind. They remained having doubts about what would be acceptable in terms of decline. One participant indicated that seeing other patients with HD was important to remind herself what she did not want to go through. She was afraid that otherwise her fearful image might fade into the background and she would then refrain from euthanasia.

*P8: “I also think I have to be faced with the facts regularly to see how sick they are and what you don’t want for yourself, so to speak. I think it’s a good thing that every now and then you see people who are further along than I am, because well, then I’ll know for sure: that is really not what I want. [...] No, I’m not going to do that.”*

#### The future is something that you would rather not think about

Participants who adopted this strategy made the poor prognosis of HD manageable by thinking about it as little as possible. They were aware of the deterioration that they would encounter. However, the thoughts of their future led to feelings that were too threatening to handle. They tried to block out this negative image of the future with the aim of living a better life in the present. In order not to think about the future, participants tried to live one day at a time, emphasized how they achieved small victories over their disease, or sought distraction by focusing on positive things in the present.

I: *“The end of life, do you ever think about that?”*

P11: *“No, I don’t really want to. [...] Well, if I start thinking about it too much, you go crazy, right? [...] No, but obviously I’m sort of burying my head in the sand a little, [laughs], [...]. Of course that helps you to keep going, yes. [ ...] Often just not think about it, about what it will be like in 5, 6, 7, 8 years from now. Easier.”*

Suppressing the realization of the future took great effort, and participants did not always seem to succeed, as can be seen in this quote from P5:

P5: *“About the future, well, I just take it one day at a time now. [...]. Because in the back of my mind I know, it can manifest itself any day and then progress. Like an express train. I know that. [ …*]. *No, I don’t want to think about it at all. [ …]. And I don’t want to talk about it either. I don’t read about it, [...], [case manager] also brings me all these booklets and leaflets [...], but look, I know what it’s like from experience. Second of all, I don’t have to read it because then I’ll immediately feel bad about it again. [ …]. And thirdly, [...] I won’t forget because the movements are there, you know? I have it anyway [laughs].”*

#### The future is something that you do not have to worry about yet

Finally, participants that used this strategy saw the future as something they did not have to worry about yet. They mentioned: you don’t know how the future will be and when the time comes, surely there will be help. In comparison with the first two strategies, where participants seemed to realize the content of their prognosis, there was room for a less negative image of the future in this strategy.

#### Hoping that the deterioration will be slow or mild

While uncertainty about the future entailed an enormous burden for some, this uncertainty gave other participants the possibility of hoping that decline would stay away or go slowly, or hoping that disease expression would be mild.

If participants had been sick for a longer time, and their disease progression had been relatively slow until then, they hoped that they would not suddenly deteriorate quickly. They were aware that their disease would worsen eventually, but they estimated that there would still be enough time to think about the future. Like P7, who believed that it might be a good idea to write her wishes down, but postponed doing so because she thought her symptoms would not get much worse in the short-term.

P7: *“I have to write these things down on paper somewhere, because it’s true that at some point you’re almost not fully accountable anymore. It can go that quickly. But anyway, I’m also a bit, uh, I may have a little more confidence now that I won’t deteriorate a lot suddenly.”*

Other participants hoped for a mild expression of HD. What was seen as mild differed per participant. For example, there were participants who greatly feared getting dementia, while P5 saw dementia as a less severe version of the disease, as this may mean that you are blissfully happy.

P5: *“When I get to that point, I may be happy, you don’t know that. [...] Look, because I know my brother [who also had HD], [...] you know with the Alzheimer’s, [...], he was very happy. Now imagine that that is the case, well you forget things here and there, [...], well okay, so maybe you are living your own life.”*

#### Having confidence that others will help you in the future

Some participants did not worry about the future because they were confident that relatives and professionals would provide adequate care when needed.

I: *“Do you have an advance directive?”*

P2: *“I don’t really know. Don’t think so. [...] I don’t have that yet. The children will take care of certain things I suppose. One of the boys. Yes, and in [location of outpatients’ clinic], you know, the center there. They also say, if you need anything, just call. And we’ll be there.”*

### Adopting different strategies

Participants could adopt more than one strategy at a time. For example, P5 tried to avoid thinking about the future, but at moments when she did not succeed in doing so, she hoped that the disease expression would be mild. Furthermore, some participants indicated that they had adopted different strategies during the course of their illness. For example, P7 mentioned that she used to think more about the future “*in the beginning when doom seemed larger*”. However, when that first doom was over, she gradually started to take each day as it came and became more indifferent of the future.

## Discussion

The aim of this study was to gain insight into the views of HD patients regarding their future and to describe how they deal with the poor prognosis of HD. The main outcome of the interviews was that while all participants seemed to be aware of their poor prognosis, they adopted different strategies to keep this knowledge ‘at a distance’ in order to keep daily life as manageable as possible. Participants dealt with the future by making arrangements for the future, by avoiding thoughts of the future and/or by not worrying about the future yet.

Participants who adopted the first strategy put more emphasis on an advance euthanasia request and less on other ways of planning future care. They seemed to think more about the conditions that would not be bearable anymore and less about the conditions that could support them at the end of their life. This finding corresponds with Booij et al. [18], who found that the majority of end-of-life wishes that HD patients had concerned euthanasia. We do know, however, that having an advance euthanasia request does not necessarily mean that the patient will request euthanasia at the end of life [24]. Above that, in the Netherlands, euthanasia or PAS is only possible when strict criteria are met and a physician can never be obliged to honour a euthanasia request. Examination of the total number of deaths by euthanasia in 2010 reveals that the percentage of HD patients is higher (12–21%) than the percentage in the general population and in patients with cancer (approximately 3 and 6% respectively) [18, 25–27]. Because of the position the Netherlands has when it comes to legislation of euthanasia, patients could be more inclined to draft up an advance euthanasia request instead of an advance directive for refusal of treatments. Therefore, drafting up an advance request for euthanasia could be typical for the Dutch situation and other countries where euthanasia and PAS are possible. However, the feeling of “*I don’t want to go through that”* or *“it’s enough”* may also be present in HD patients in other countries. It is possible that these patients express this same feeling in other advance directives.

In adopting the second strategy, participants would rather avoid thinking about the future. Living in the present seems to be challenging enough. Participants are surviving day by day and are proud when they succeed in doing so. Furthermore, avoidance is recognized as an emotion-focused coping strategy [28]. Focusing attention on something less negative than the prospect of future deterioration allows individuals to proceed with their daily lives. Interestingly, avoidance was also found as a strategy in caregivers of patients with HD when it concerned thinking or talking about the future [29].

In the third strategy, participants felt it was not yet necessary to think about the future. Again, this way of thinking could be motivated by several factors. First, it is possible that patients are too optimistic about the prognosis because they can no longer monitor their own functioning and symptoms adequately. This lack of awareness of functional decline could be due to a neurological deficit [30]. For example, one participant was completely unaware of her situation even though motor symptoms were clearly present and she had been receiving HD-related care for several years. Second, the need for hope could be an important factor in this strategy. Hope has been shown to play an important role in palliative care [31, 32]. In this study, hope was directed towards a slow course or mild manifestation of the illness; it did not concern a cure in the near future.

The findings of our study showed similarities to studies on other neurodegenerative diseases, such as dementia, Parkinson’s disease, Progressive Supranuclear Palsy, Motor Neurone disease and Multiple Sclerosis [9, 11]. Both Clarke et al. [9] and De Boer et al. [11] found that while some patients wished to plan for the future, others preferred to take each day as it comes and avoid worrying about the future. In our study, we also found participants who wished to plan for the future as opposed to others who did not make such plans. However, in our study this was less of a contradiction as it seemed, because participants who wanted to plan for the future usually returned to their daily lives once they had made their arrangements for the future. For participants who drafted up an euthanasia request, their preparation for the future seemed primarily to consist of getting the reassurance that they do not have to endure the disease until its very end.

What our study furthermore adds concerns the variation within the group of people who prefer to take each day as it comes and avoid worrying about the future. Some participants had a view of the future as something so frightful that they desperately avoided thinking about it and tried to focus exclusively on the present. In contrast, other participants viewed the future less negatively and saw no need to think about the future at this time. A follow-up study could further investigate whether this latter strategy is typical for HD patients. Although aspects of HD that are characteristic of the disease generally caused participants in our study to take a more negative view of their future prospects, a less negative view of the future sometimes resulted as well. For example, the prospect of a long disease trajectory made P7 less anxious for the future and made her put her plans for drafting up an advance directive on hold. In another example, familiarity with the disease in the family of P5 appeared to be a motivation to hope for a mild manifestation of the disease, like she saw in her relative.

As mentioned before, it is recommended that ACP conversations with HD patients should begin in an early stage, because of the potential decline in decision-making capacity and communication [9–11]. ACP is a dynamic and continuous process consisting of multiple returning conversations about the future. However, the question arises to what extent patients are receptive to these returning conversations. In order to keep daily life as manageable as possible, we saw that participants tried to keep their future at a distance and preferred to speak about their life in the present. When they did want to think and talk about their future, they wanted to make arrangements, usually for euthanasia, and then return to their daily life. They seemed to see ACP as a one-time thing, which is different from how ACP is intended. Hence, there seems to be a contrast between patient preferences and the recommendations from the theory of ACP. This could raise a dilemma for health-care providers in daily practice. We recommend that future research should examine how health-care providers experience this discrepancy in daily practice and how they deal with this potential dilemma in working with patients with HD.

Because this was a cross-sectional qualitative study and we interviewed only patients that received outpatient care, we do not know whether the way HD patients deal with their future remains stable over time. Patients who are more advanced in their illness may think differently about their future than the patients we interviewed. We recommend following patients over a longer period of time to examine the extent to which the passage of time alters the way patients think about the future and future care, and the way they deal with their future prospects.

### Strengths and limitations

A strength of this study is that we conducted 12 face-to-face interviews with patients. Having face-to-face interviews with patients made thorough exploration of their individual perspectives possible. All participants were able to express their opinions and thoughts, even though sometimes it took some time or effort. Furthermore, we could obtain a variety of perspectives as there was variation in the group in terms of demographics (e.g. age, family living conditions). However, it is possible that we did not find sufficient variation in all aspects, also due to limited sample size. For example, the group of participants was homogeneous regarding religious beliefs; all patients indicated that they were either not religious or not active in their religion. Therefore, we may have missed certain perspectives. Another limitation is a selection bias that may have occurred. On the one hand, patients might not have wanted to participate in such a study when they did not want to think or talk about HD or their future; on the other hand, nurses might have been inclined to suggest participation only to patients who could talk easily about these subjects. However, the responses given in the interviews indicate that both types of patients participated in the study.

## Conclusions

This interview study has provided valuable insight into the views of HD patients regarding their future and planning future care, and how patients deal with the prognosis of HD. A contrast seems to exist between the ACP recommendation to discuss goals and wishes for the future and the tendency of patients to keep the future at a distance. Health-care providers may consider starting conversations in the present as well by exploring current everyday struggles and victories, personal values and life goals. From there, conversations may continue about the future and preferences for future care. Anticipating the future and ACP involves more than euthanasia alone, so alternatives should be discussed as well.

## Supplementary Information

**Additional file 1: Supplementary file 1** Topic list for patients with HD.

## Data Availability

The datasets used during the current study are available from the corresponding author on reasonable request.

## Abbreviations

ACP: Advance care planning
HD: Huntington’s disease
PAS: Physician assisted suicide

## References

[1] Roos RA (2010). Huntington's disease: a clinical review. Orphanet J Rare Dis..

[2] Wheelock VL, Tempkin T, Marder K, Nance M, Myers RH, Zhao H (2003). Predictors of nursing home placement in Huntington disease. Neurology..

[3] Simpson SA (2007). Late stage care in Huntington's disease. Brain Res Bull..

[4] Klager J, Duckett A, Sandler S, Moskowitz C (2008). Huntington's disease: a caring approach to the end of life. Care Manag J..

[5] Downing NR, Goodnight S, Chae S, Perlmutter JS, McCormack M, Hahn E (2018). Factors Associated With End-of-Life Planning in Huntington Disease. Am J Hosp Palliat Care..

[6] Rietjens JAC, Sudore RL, Connolly M, van Delden JJ, Drickamer MA, Droger M (2017). Definition and recommendations for advance care planning: an international consensus supported by the European Association for Palliative Care. Lancet Oncol..

[7] Brinkman-Stoppelenburg A, Rietjens JA, van der Heide A (2014). The effects of advance care planning on end-of-life care: a systematic review. Palliat Med..

[8] Zielonka D, Marinus J, Roos RA, De Michele G, Di Donato S, Putter H (2013). The influence of gender on phenotype and disease progression in patients with Huntington's disease. Parkinsonism Relat Disord..

[9] Clarke G, Fistein E, Holland A, Tobin J, Barclay S, Barclay S (2018). Planning for an uncertain future in progressive neurological disease: a qualitative study of patient and family decision-making with a focus on eating and drinking. BMC Neurol..

[10] Verschuur EML, Geense WW, Adriaansen MJM (2020). Support Need Among Patients with Huntington Disease in the Netherlands: Results of a Focus Group Study. Nur Primary Care..

[11] De Boer M, Dröes R-M, Jonker C, Eefsting J, Hertogh C (2012). Thoughts on the Future: The Perspectives of Elderly People with Early-Stage Alzheimer's Disease and the Implications for Advance Care Planning. Ajob Primary Research..

[12] Ghielen I, Rutten S, Boeschoten RE, Houniet-de Gier M, van Wegen EEH, van den Heuvel OA (2019). The effects of cognitive behavioral and mindfulness-based therapies on psychological distress in patients with multiple sclerosis, Parkinson's disease and Huntington's disease: Two meta-analyses. J Psychosom Res..

[13] Regan L, Preston NJ, Eccles FJR, Simpson J (2019). The views of adults with neurodegenerative diseases on end-of-life care: a metasynthesis. Aging Ment Health..

[14] Domaradzki J (2015). The Impact of Huntington Disease on Family Carers: a Literature Overview. Psychiatr Pol..

[15] Carlozzi NE, Boileau NR, Paulsen JS, Perlmutter JS, Lai JS, Hahn EA (2019). End-of-life measures in Huntington disease: HDQLIFE Meaning and Purpose, Concern with Death and Dying, and End of Life Planning. J Neurol..

[16] Aubeeluck A, Buchanan H (2007). The Huntington's disease quality of life battery for carers: reliability and validity. Clin Genet..

[17] Ready RE, Mathews M, Leserman A, Paulsen JS (2008). Patient and caregiver quality of life in Huntington's disease. Mov Disord..

[18] Booij SJ, Tibben A, Engberts DP, Marinus J, Roos RA (2014). Thinking about the end of life: a common issue for patients with Huntington's disease. J Neurol..

[19] Adams WC, Newcomer KE, Hatry HP, Wholey JS (2015). Conducting Semi-Structured Interviews. Handbook of Practical Program Evaluation.

[20] Galletta A, Cross WE. Mastering the Semi-Structured Interview and Beyond. From Research Design to Analysis and Publication. New York: NYU Press; 2013.

[21] Patton MQ (1990). Qualitative evaluation and research methods.

[22] Birks M, Chapman Y, Francis K (2008). Memoing in qualitative research: Probing data and processes. J Res Nurs..

[23] Phillippi J, Lauderdale J (2018). A Guide to Field Notes for Qualitative Research: Context and Conversation. Qual Health Res..

[24] Bolt EE, Pasman HR, Deeg DJ, Onwuteaka-Philipsen BD (2016). From Advance Euthanasia Directive to Euthanasia: Stable Preference in Older People?. J Am Geriatr Soc..

[25] Gubert C, Renoir T, Hannan AJ (2020). Why Woody got the blues: The neurobiology of depression in Huntington's disease. Neurobiol Dis..

[26] Onwuteaka-Philipsen BD, Brinkman-Stoppelenburg A, Penning C, de Jong-Krul GJ, van Delden JJ, van der Heide A (2012). Trends in end-of-life practices before and after the enactment of the euthanasia law in the Netherlands from 1990 to 2010: a repeated cross-sectional survey. Lancet..

[27] Eddy CM, Parkinson EG, Rickards HE (2016). Changes in mental state and behaviour in Huntington's disease. Lancet Psychiatry..

[28] Lazarus RS, Folkman S (1984). Stress, appraisal, and coping.

[29] Lowit A, van Teijlingen ER (2005). Avoidance as a strategy of (not) coping: qualitative interviews with carers of Huntington's Disease patients. BMC Fam Pract..

[30] Sitek EJ, Thompson JC, Craufurd D, Snowden JS (2014). Unawareness of deficits in Huntington's disease. J Huntingtons Dis..

[31] Olsman E, Leget C, Onwuteaka-Philipsen B, Willems D (2014). Should palliative care patients' hope be truthful, helpful or valuable? An interpretative synthesis of literature describing healthcare professionals' perspectives on hope of palliative care patients. Palliat Med..

[32] Daneault S, Lussier V, Mongeau S, Yelle L, Cote A, Sicotte C (2016). Ultimate journey of the terminally ill: Ways and pathways of hope. Can Fam Physician..

