# Supplementary material for: Gaining insight into the views of outpatients with Huntington’s disease regarding their future and the way they deal with their poor prognosis: a qualitative study
Source: BMC Palliat Care. 2021 Jan 12;20:12. doi: 10.1186/s12904-020-00706-x (PMC7802313; doi:10.1186/s12904-020-00706-x)
Supplement: Supplementary file 1 — Additional file 1: Supplementary file 1 Topic list for patients with HD. [file 12904_2020_706_MOESM1_ESM.doc]

**Supplementary file 1: Topic list for patients with HD**

**Background**

Current living situation

When diagnosed with HD

Experience with HD in the family

**Quality of life**

Present quality of life

What is important in life

**Future**

Thoughts and attitudes towards the future

Uncertainty/ambiguity regarding these thoughts and attitudes

Professional care in the future

Discussing the future with others

**End of life**

Thoughts and attitudes towards the end of life

Uncertainty/ambiguity regarding these thoughts and attitudes

Professional care at the end of life

Discussing the end of life with others

**(Future) medical treatment**

Thoughts and attitudes towards (future) medical treatment

Uncertainty/ambiguity regarding these thoughts and attitudes

Presence of advance directives

Discussing (future) medical treatment and advance directives with others

**The possibility of no longer wanting to live**

Thoughts and attitudes towards the possibility of no longer wanting to live

Uncertainty/ambiguity regarding these thoughts and attitudes

Presence of an advance euthanasia request

Discussing the possibility of no longer wanting to live with others

**Religion/spirituality**

Role of religion or spirituality in thoughts and attitudes towards aforementioned topics
